# Penalized Multi-Way Partial Least Squares for Smooth Trajectory Decoding from Electrocorticographic (ECoG) Recording

**DOI:** 10.1371/journal.pone.0154878

**Published:** 2016-05-19

**Authors:** Andrey Eliseyev, Tetiana Aksenova

**Affiliations:** CEA-LETI-CLINATEC, Grenoble, France; Shanghai Jiao Tong University, CHINA

## Abstract

In the current paper the decoding algorithms for motor-related BCI systems for continuous upper limb trajectory prediction are considered. Two methods for the smooth prediction, namely Sobolev and Polynomial Penalized Multi-Way Partial Least Squares (PLS) regressions, are proposed. The methods are compared to the Multi-Way Partial Least Squares and Kalman Filter approaches. The comparison demonstrated that the proposed methods combined the prediction accuracy of the algorithms of the PLS family and trajectory smoothness of the Kalman Filter. In addition, the prediction delay is significantly lower for the proposed algorithms than for the Kalman Filter approach. The proposed methods could be applied in a wide range of applications beyond neuroscience.

## Introduction

The Brain-Computer Interface (BCI) is a system for converting the brain’s neural activity into commands for external devices [1]. Motor-related BCI aims at improving the quality of life of individuals with severe motor disabilities [2]. Paralyzed or handicapped persons get the opportunity for mental control of wheelchairs, robotic arms, exoskeletons, etc. [3–8]. Besides that, motor-related BCI systems can be applied for rehabilitation purposes [9, 10].

Different methods of acquiring the brain’s neural activity are currently used for BCI. Among the non-invasive approaches are electroencephalography (EEG) [11, 12], magnetoencephalography (MEG) [7, 13], functional magnet resonance imaging (fMRI) [14, 15], functional near-infrared spectroscopy (fNIRS) [16, 17], etc. In the case of invasive methods such as electrocorticography (ECoG) [18–22] and microelectrode arrays (ME) recordings [23, 24], the electrodes are implanted under the cranial bones. Whereas ME record intra-cortical neural activity, ECoG electrodes are placed on the surface of the brain. A chronic ECoG-recording implant, appropriate for a long-term use, has recently been reported [25]. Although non-invasive techniques ensure better safety, the invasive methods allow superior quality of the recordings, providing high spatial and temporal resolution of the signals [18]. Decoding of hand’s continuous trajectories from subdural and epidural ECoG recordings was reported recently [22–24, 26–29].

In this paper, the task of reconstructing continuous 3D trajectories of the subject’s hand from ECoG recordings is considered. This problem was studied in both monkeys and humans [22, 29–31]. A set of approaches for trajectory decoding from neuronal recordings were proposed [22–24, 28]. A common approach consists in extraction of informative features from spatial [32], frequency [33], or/and temporal [34] domains of brain signal. Decoder is identified from the trajectory information (response variable) and neural activity features (exploratory variable). Many standard methods for model identification from experimental data are designed for vector exploratory variables which generally represent only one domain (modality) of analysis [26, 35–37]. If analysis in one domain does not provide satisfactory results, several modalities could be combined sequentially in this paradigm. A tensor-based (Multi-Way) approach is intended for simultaneous treatment of several domains. Multi-electrodes brain signals recording is mapped to the spatial-temporal-frequency space [38] representing single time epoch by a cube (a third-order tensor). All cubes are stored in a tensor of observations (a fourth-order tensor). Tensor (multi-way array) data representation is convenient, since it save natural structure of the data. Detailed review of the tensor data representation as well as the tensor analysis is given in [39]. Multi-Way Analysis is reported as an efficient tool for the decoder identification [22, 40, 41].

The peculiarity of the tensor data analysis is their high dimension. The algorithms of the Partial Least Squares (PLS) regression family are known to be particularly appropriate for high dimension tasks [42]. PLS projects both exploratory and response variables to low dimensional spaces of latent variables. Contrary to the widespread Principal Component Analysis (PCA) [43], which projects only explanatory variables, both explanatory and response variables are taken into account by the PLS for projectors identification. Thus, the PLS provides projectors correlated to the task. However, the generic PLS approach is vector oriented and can be applied only for the tensors unfolded to the vectors (Unfolded PLS) [44]. To exploit the advantages of tensor data representation, generalizations of the PLS for the tensor data were invented (N-Way PLS [45], Higher Order PLS [40], etc.) and were used in BCI systems [40, 46, 47].

The smoothness of the predicted trajectory is an important property of motor-related BCI systems [29, 48, 49]. The particular importance of smoothness was reported for control of such BCI effectors as wheelchairs [50] and cars [51]. There are two main approaches to manage the problem of a smooth prediction. The first one consists in the smoothing post-processing of the predicted trajectories [29]. However, this post-processing increases the system’s latency, depending on the required level of smoothness, the decision rate of the system, etc. Another solution consists in identifying the model, for which the smoothness of the predicted trajectories is an intrinsic property. A Kalman Filter (KF) [52] is used in BCI systems to generate smooth trajectories [53, 54]. However, the method is not adjusted for the high dimensional data due to the application of the matrix inversion operation in it. Moreover, the Kalman Filter weights the current prediction with the previous one, which also brings a latency to the system. Minimal system latency is an essential requirement for the comfortable use of real-time BCI systems [55]. Improvement of the prediction smoothness with minimal supplementary delay is the particular goal of the article.

In this paper, a new penalization approach is proposed to improve neural decoding smoothness. By means of penalization, one can impose different penalties related to the particular tasks. The paper novelty resides in two new ways of penalization (new penalization terms), which particularly address solution smoothness. Namely, Sobolev Penalized Multi-Way PLS (SNPLS) and Polynomial Penalized Multi-Way PLS (PNPLS), are proposed. They combine tensor representation of the data with the possibility to control the level of the smoothness of the predicted trajectories. In these methods, the smoothness is not provided by the post-processing or weighting with previous predictions, but is an internal parameter of the identified model. Thus, no supplementary delay is introduced due to post-processing of the response. In the current paper, a set of state of the art BCI methods, namely Kalman Filter, generic PLS, and Multi-Way PLS (NPLS), are considered and compared with the proposed SNPLS and PNPLS on the set of publicly available ECoG recordings. In addition, response latencies are investigated and compared for the proposed approach and state of the art methods.

The proposed methods could be applied in a wide range of applications beyond neuroscience.

### Prediction performance evaluation

Different criteria for performance evaluation have been applied in BCI studies [26, 29, 40, 56]. In this paper, Pearson correlation (*r*), Root Mean Squares Error (RMSE), and Mean Absolute Error (MAE) are used to compare the methods. A shortcoming of the above criteria is that they do not reflect the smoothness of the predicted trajectory. They are based on the sum of squares/absolute values of residuals which temporal sequence is not taken into account. Mean Absolute Differential Error (MADE) is a way to assess both prediction accuracy and smoothness [29]. Hence MADE is included to the list of criteria for performance evaluation.

The Pearson correlation is a commonly used criterion [22, 26, 30, 46] for the continued-value trajectories decoding,

$$r=\text{correlation}\left(y,\hat{y}\right),$$ (1)

Where **y** = (*y*(*t*_1_),…,*y*(*t*_*N*_))^*T*^ = (*y*_1_,…,*y*_*N*_)^*T*^ and $\hat{y}={\left(\hat{y}\left(t_{1}\right),\ldots ,\hat{y}\left(t_{N}\right)\right)}^{T}={\left({\hat{y}}_{1},\ldots ,{\hat{y}}_{N}\right)}^{T}$ are the observed and predicted trajectories. RMSE represents the *L*_2_-norm distance between the predicted and observed trajectories [22, 29, 40]:

$$\text{RMSE}={\left\|y-\hat{y}\right\|}_{2}/{\left\|y-\bar{y}\right\|}_{2},\;{\left\|y\right\|}_{2}=\sqrt{\sum_{i=1}^{N}y_{i}^{2}},\;\bar{y}=\sum_{i=1}^{N}y_{i}/N.$$ (2)

Less sensitive to the outliers, MAE [29, 56] is based on the *L*_1_-norm distance:

$$\text{MAE}={\left\|y-\hat{y}\right\|}_{1}/{\left\|y-\bar{y}\right\|}_{1},\;{\left\|y\right\|}_{1}=\sum_{i=1}^{N}\left|y_{i}\right|.$$ (3)

MADE criterion compares derivatives **y**' and y^':

MADE=‖y'−y^'‖1/‖y'−y¯'‖1,y¯'=∑i=1Nyi'/N. (4)

MADE characterizes the prediction smoothness [29] and, being based on the *L*_1_-norm, is robust to the outliers.

## Methods

### Kalman Filter

The KF [52, 57] is a vector-oriented method, which recursively operates on streams of input data to produce an estimate of the underlying system state. KF is widely used to decode the neural activity in BCIs [53, 58–61]. One advantage of KF is the smoothness of the prediction in time [53, 59]. Thus, KF was applied for prediction of the hand’s trajectory from the brain’s neuronal activity, such as ECoG [60] and spikes [53, 61]. Brain activity data $x_{k}\in {ℜ}^{n}$, observed at the moment *t*_*k*_, are used to predict the system state $y_{k}\in {ℜ}^{m}$ (*m* = 9 represents 3D coordinates, velocity, and acceleration of the hand at the moment *t*_*k*_). The generative model of the KF is formulated as a linear dependency between the brain activity and system state: **x**_*k*_ = **H**_*k*_**y**_*k*_+**q**_*k*_, where $H_{k}\in {ℜ}^{n\times m}$ is a matrix of linear coefficients, and $q_{k}\sim N\left(0,Q_{k}\right)$ is the zero-mean noise of the observation, $Q_{k}\in {ℜ}^{n\times n}$. The model expects that the system states are linearly propagated in time: **y**_*k*+1_ = **A**_*k*_**y**_*k*_+**w**_*k*_, where $A_{k}\in {ℜ}^{m\times m}$ is a matrix of linear coefficients, $w_{k}\sim N\left(0,W_{k}\right)$, $W_{k}\in {ℜ}^{m\times m}$. If matrices **H**_*k*_ = **H**, **A**_*k*_ = **A**, **Q**_*k*_ = **Q**, and **W**_*k*_ = **W** are constants in time, they can be estimated from the training data $X=\left(x_{1},\ldots ,x_{N}\right)\in {ℜ}^{n\times N}$, $Y=\left(y_{1},\ldots ,y_{N}\right)\in {ℜ}^{m\times N}$ by means of the least squares:

$$A=Y_{2}Y_{1}^{T}{\left(Y_{1}Y_{1}^{T}\right)}^{-1},\;H=XY^{T}{\left(YY^{T}\right)}^{-1},\;W=\left(Y_{2}-AY_{1}\right){\left(Y_{2}-AY_{1}\right)}^{T}/\left(N-1\right),$$

$$Q=\left(X-\mathrm{HY}\right){\left(X-\mathrm{HY}\right)}^{T}/N,\;Y_{1}=\left(y_{1},\ldots ,y_{N-1}\right),\;Y_{2}=\left(y_{2},\ldots ,y_{N}\right).$$ (5)

The KF algorithm consists of *a priori* and *a posteriori* steps. In the first step, the method *a priori* estimates the state of the system as ${\hat{y}}_{k}^{-}=A{\hat{y}}_{k-1}$. In the second step, *a posteriori* estimation ${\hat{y}}_{k}$ is found as a linear combination of the *a priori* estimation and the weighted difference between the observed and predicted signals:

$${\hat{y}}_{k}={\hat{y}}_{k}^{-}+K_{k}\left(x_{k}-H{\hat{y}}_{k}^{-}\right).$$ (6)

Here $K_{k}=P_{k}^{-}H^{T}{\left(HP_{k}^{-}H^{T}+Q\right)}^{-1}$ is the gain matrix, $P_{k}^{-}=AP_{k-1}A^{T}+W$ is the *a priori* estimate error, and $P_{k}=\left(I-K_{k}H\right)P_{k}^{-}$ is the *a posteriori* estimate error [52]. More detailed information about the KF can be found in [57].

### Generic PLS

A linear regression model between the response variable $y\in {ℜ}^{m}$ and the explanatory variable $x\in {ℜ}^{n}$ is represented as $E\left[y|x\right]=\sum_{i=1}^{n}\beta_{i}x_{i}=\mathrm{Bx}$, where $B=\left(\beta_{1},\ldots ,\beta_{n}\right)\in {ℜ}^{m\times n}$, **x** = (*x*_1_,…,*x*_*N*_)^*T*^ [62]. For the data set **X** = (**x**_1_,…,**x**_*N*_)^*T*^, **Y** = (**y**_1_,…,**y**_*N*_)^*T*^, the Ordinary Least Squares (OLS) gives [63]:

$$B=\underset{B}{\arg\;\min}\sum_{i=1}^{N}{\left\|y_{i}-Bx_{i}\right\|}_{2}^{2}=\underset{B}{\arg\;\min}{\left\|Y-\mathrm{BX}\right\|}_{2}^{2}.$$ (7)

However, the OLS procedure is unstable for the case of the high dimension task [63]. Partial Least Squares regression is a statistical method for linear vector-based data analysis [42]. Due to its efficient dimension reduction technique even in the case of highly correlated variables, PLS is particularly appropriate for the high dimensional data. The PLS algorithm iteratively estimates a linear relation between the matrices of observations of input and output variables $X\in {ℜ}^{N\times n}$ and $Y\in {ℜ}^{N\times m}$: **Y** = **XB** + **D**, where $B\in {ℜ}^{n\times m}$ is the matrix of linear coefficients, and $D\in {ℜ}^{N\times m}$ is the matrix of noise. On each iteration, **X** and **Y** are projected in low dimension spaces (latent variables) in such a way as to explain the maximum of their variation simultaneously: **X** = **TP**^*T*^ + **E**, **Y** = **UQ**^*T*^ + **F**, where $T=\left[t_{1},\ldots ,t_{F}\right]\in {ℜ}^{N\times F}$ and $U=\left[u_{1},\ldots ,u_{F}\right]\in {ℜ}^{N\times F}$ are the matrix of the latent variables (scores), $P=\left[p_{1},\ldots ,p_{F}\right]\in {ℜ}^{n\times F}$ and $Q=\left[q_{1},\ldots ,q_{F}\right]\in {ℜ}^{m\times F}$ are the matrix of the loading vectors (projectors), **E** and **F** are residual matrices, and *F* is the number of iterations (factors). Estimation of the factors number *F* can be done through the cross-validation procedure [64]. A detailed description of the PLS approach can be found in [42].

The PLS regression was applied in the BCI systems for hand trajectory reconstruction [22, 26, 40].

### Multi-way PLS

Multi-way (N-way) PLS is a generalization of the PLS method to the tensor input/output data [45]. Tensors (multi-way arrays) allow the representation of data of a high order. Vectors and matrices are tensors of orders one and two, respectively. In this paper, a tensor is denoted as $\underline{X}\in {ℜ}^{I_{1}\times \ldots \times I_{n}}$, where *n* is the order of the tensor. More information about the tensors can be found in [39]. NPLS inherits the robustness of PLS and allows simultaneous analysis of data in several domains (e.g. time-frequency-space). Examples of the application of multi-way tensor-based methods in BCI research are given in [40, 46, 65–67].

Similarly to PLS regression, NPLS builds the linear relation between the dependent and independent tensors of observations $\underline{X}\in {ℜ}^{N\times I_{1}\times \ldots \times I_{n}}$ and $\underline{Y}\in {ℜ}^{N\times J_{1}\times \ldots \times J_{m}}$. The model is constructed iteratively by projection of the tensors to the space of latent variables:

$$\underline{X}=\sum_{f=1}^{F}t_{f}\circ w_{1}^{f}\circ \ldots \circ w_{n}^{f}+\underline{E},\;\underline{Y}=\sum_{f=1}^{F}u_{f}\circ q_{1}^{f}\circ \ldots \circ q_{m}^{f}+\underline{F},\;u_{f}=T_{f}B_{f},$$ (8)

where the operator “°” is the outer product [39], $T_{f}=\left[t_{1},\ldots ,t_{f}\right]\in {ℜ}^{N\times f}$ and $U_{f}=\left[u_{1},\ldots ,u_{f}\right]\in {ℜ}^{N\times f}$ are the matrices of the latent variables after *f* = 1,…,*F* iterations, $w_{i}^{f}\in {ℜ}^{I_{i}}$ and $q_{j}^{f}\in {ℜ}^{J_{j}}$ are the projection vectors, **B**_*f*_ is the matrix of the linear coefficients, and **E** and **F** are the residual tensors.

In contrast to the OLS approach, NPLS, as well as PLS, is a biased estimator. However, it provides the insensitivity of the predictive model to ill-conditioning and noise [68].

### Penalized regression

Modification of the cost function (optimization functional) in the model identification procedure could be used to improve the prediction/regression properties. Among other reasons, penalizations are widely applied to achieve desirable characteristics (sparsity, robustness, etc.). Additional terms are added to the optimization task, basically performing the standard *L*_1_-norm or *L*_2_-norm optimization. For linear regression *E*[**y**|**x**] = **Bx** the least squares estimation is given in (7). To impose the additional restrictions, the regularization (penalization) term is introduced [69–74].

$$B=\underset{B}{\arg\;\min}\;{\left\|Y-\mathrm{BX}\right\|}_{2}^{2}+P\left(Y,B,X\right),$$ (9)

$P_{L_{1}}\left(B,\lambda \right)=\lambda {\left\|B\right\|}_{1}^{2}$ (LASSO) and $P_{L_{2}}\left(B,\lambda \right)=\lambda {\left\|B\right\|}_{2}^{2}$ (RIDGE) are the most frequently used, *λ* ≥ 0 represents the penalization parameter. Whereas the $P_{L_{1}}$ penalization encourages sparse solution (9), the $P_{L_{2}}$ penalization reduces the variation of the coefficients **B**, which increases the robustness of the model [71]. In addition, the $P_{L_{2}}$ penalization as well as Fused LASSO [75] are often applied to improve the smoothness of the coefficients **B** [70, 73, 74]. However, neither $P_{L_{1}}$ nor $P_{L_{2}}$ penalizations improve the smoothness of prediction of **y**(t) analyzing the data flow **x**(t). In this paper, we propose two penalizations to improve the smoothness of the continuous trajectories prediction.

#### SNPLS

The Sobolev NPLS method is based on the use of the Sobolev norm *W*_*s*_ [76] instead of the *L*_2_-norm in the optimization task:

$$W_{s}\;\text{norm}:\;{\left\|\phi \right\|}_{w_{s}}^{2}={\left\|\phi \right\|}_{2}^{2}+{\left\|\phi^{\left(s\right)}\right\|}_{2}^{2}.$$ (10)

Here *ϕ* and *ϕ*^*(s)*^ are a function and its *s*-th derivation, respectively. Optimization in Sobolev space corresponds to the optimization problem (9), where the penalization term is added with the parameter *λ* ≥ 0 to control the influence of the derivative part:

$$B=\underset{B}{\arg\;\min}\sum_{i=1}^{N}{\left\|y_{i}-Bx_{i}\right\|}^{2}+\lambda \sum_{i=2}^{N}{\left\|Bx^{\left(s\right)}{}_{i}\right\|}^{2}.$$ (11)

The problem can be represented in the matrix form:

$$B=\underset{B}{\arg\;\min}\;{\left\|Y-\mathrm{BX}\right\|}^{2}+{\left\|\sqrt{\lambda }BX^{\left(s\right)}\right\|}^{2}=\underset{B}{\arg\;\min}\;{\left\|\tilde{Y}-B\tilde{X}\right\|}^{2},$$ (12)

where $\tilde{X}=\left[\begin{array}{c} X\\ \sqrt{\lambda }X^{\left(s\right)} \end{array}\right]$, $\tilde{Y}=\left[\begin{array}{c} Y\\ 0 \end{array}\right]$.

The optimization problem (12) corresponds to the regression estimation task with the matrices of independent and dependent variables $\tilde{X}$ and $\tilde{Y}$, respectively. Moreover, for the tensor case, the same approach can be applied to obtain the tensors $\underline{\tilde{X}}$ and $\underline{\tilde{Y}}$. To identify the regression between the tensors $\underline{\tilde{X}}$ and $\underline{\tilde{Y}}$, we use the NPLS algorithm, since it is suitable for tensor-based variables and has demonstrated its efficiency for BCI tasks.

#### PNPLS

Polynomial Penalized NPLS considers the optimization problem:

$$B=\underset{B}{\arg\;\min}\sum_{i=1}^{N}{\left\|y_{i}-Bx_{i}\right\|}^{2}+\lambda \sum_{i=l+1}^{N-l}{\left\|g_{p,l}\left(x_{i},B\right)\right\|}^{2},$$

$$g_{p,l}\left(x_{i},B\right)=Bx_{i}-B\left(\begin{array}{c} P_{p,\left(x_{i-l}^{1},\ldots ,x_{i+l}^{1}\right)}\left(x_{i}^{1}\right)\\ \cdots \\ P_{p,\left(x_{i-l}^{n},\ldots ,x_{i+l}^{n}\right)}\left(x_{i}^{n}\right) \end{array}\right)=Bx_{i}-BP_{p,\left(x_{i-l},\ldots ,x_{i+l}\right)}\left(x_{i}\right)=Bx_{i}-B{\overset{⌢}{x}}_{i},$$ (13)

where $x_{i}={\left(x_{i}^{1},\ldots ,x_{i}^{n}\right)}^{T}$, and $P_{p,\left(x_{i-l},\ldots ,x_{i+l}\right)}\left(\cdot \right)$ is a polynomial function of the power *p*, which provides the minimal least squares approximation of the set of points (*x*_*i-l*_,…,*x*_*i+l*_), ${\overset{⌢}{x}}_{i}=P_{p,\left(x_{i-l},\ldots ,x_{i+l}\right)}\left(x_{i}\right)$. The procedure of $\overset{⌢}{x}$ identification can be represented as a result of the power *p* polynomial filtration in a sliding window of size (2*l* + 1), where polynomial coefficients are fitted independently for each window.

In the matrix notation, the optimization problem is represented as

$$B=\underset{B}{\arg\;\min}{\left\|Y-\mathrm{BX}\right\|}^{2}+\lambda {\left\|\mathrm{BX}-B\overset{⌢}{X}\right\|}^{2},$$ (14)

Penalizing the difference between the results of application of the model **B** to the initial (**X**) and “smoothed” ($\overset{⌢}{X}$) explanatory variables, this algorithm is looking for the model which provides similar results being applied to the original and “smoothed” data.

For the new variables $\tilde{X}$ and $\tilde{Y}$:

$$B=\underset{B}{\arg\;\min}{\left\|\tilde{Y}-B\tilde{X}\right\|}^{2},\;\tilde{X}=\left[\begin{array}{c} X\\ \sqrt{\lambda }\left(X-\overset{⌢}{X}\right) \end{array}\right],\;\tilde{Y}=\left[\begin{array}{c} Y\\ 0 \end{array}\right].$$ (15)

Similar to (12), the task (15) corresponds to the problem of regression estimation with the matrix of the explanatory and response variables $\tilde{X}$ and $\tilde{Y}$. It can also be generalized to the case of the tensors $\underline{\tilde{X}}$ and $\underline{\tilde{Y}}$. The NPLS algorithm is used for the following regression estimation.

Both SNPLS and PNPLS algorithms improve solutions without increasing the complexity of the optimization problem (sum-of-squares optimization).

## Application

### Evaluation and comparison

The BCI problem of the reconstruction of 3D trajectories of a monkey hand from its ECoG brain activity is considered to evaluate the accuracy and smoothness of the proposed approaches. Two proposed methods (SNPLS and PNPLS) are evaluated and compared to three state-of-the-art methods (Kalman Filter, generic PLS, and tensor-based NPLS).

**Fig 1:**
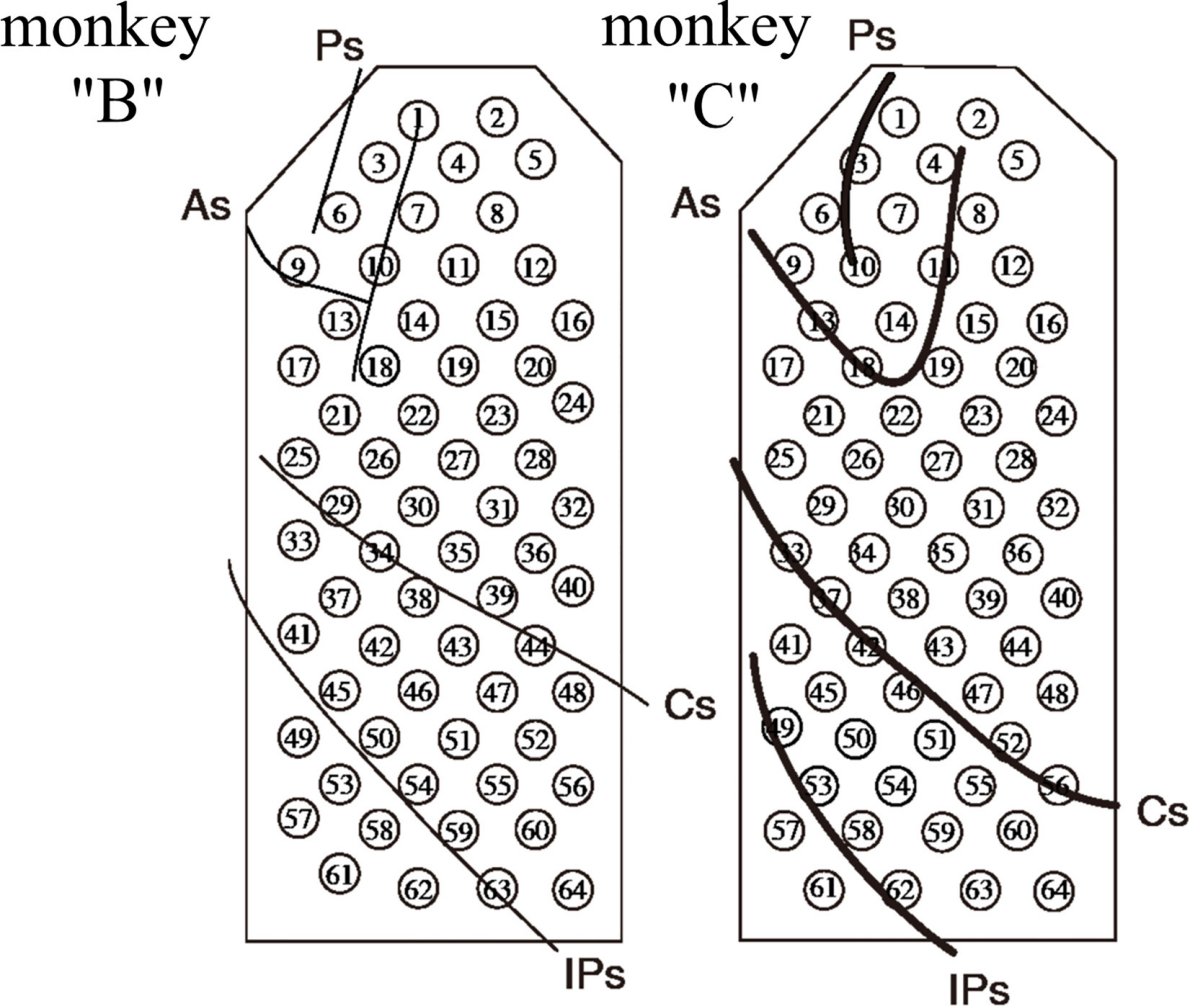
The schemes of the implantations. 64 electrodes were implanted in the epidural space of the left hemispheres of two monkeys. Ps: principal sulcus; As: arcuate sulcus; Cs: central sulcus; IPs: intraparietal sulcus [22].

**Fig 2:**
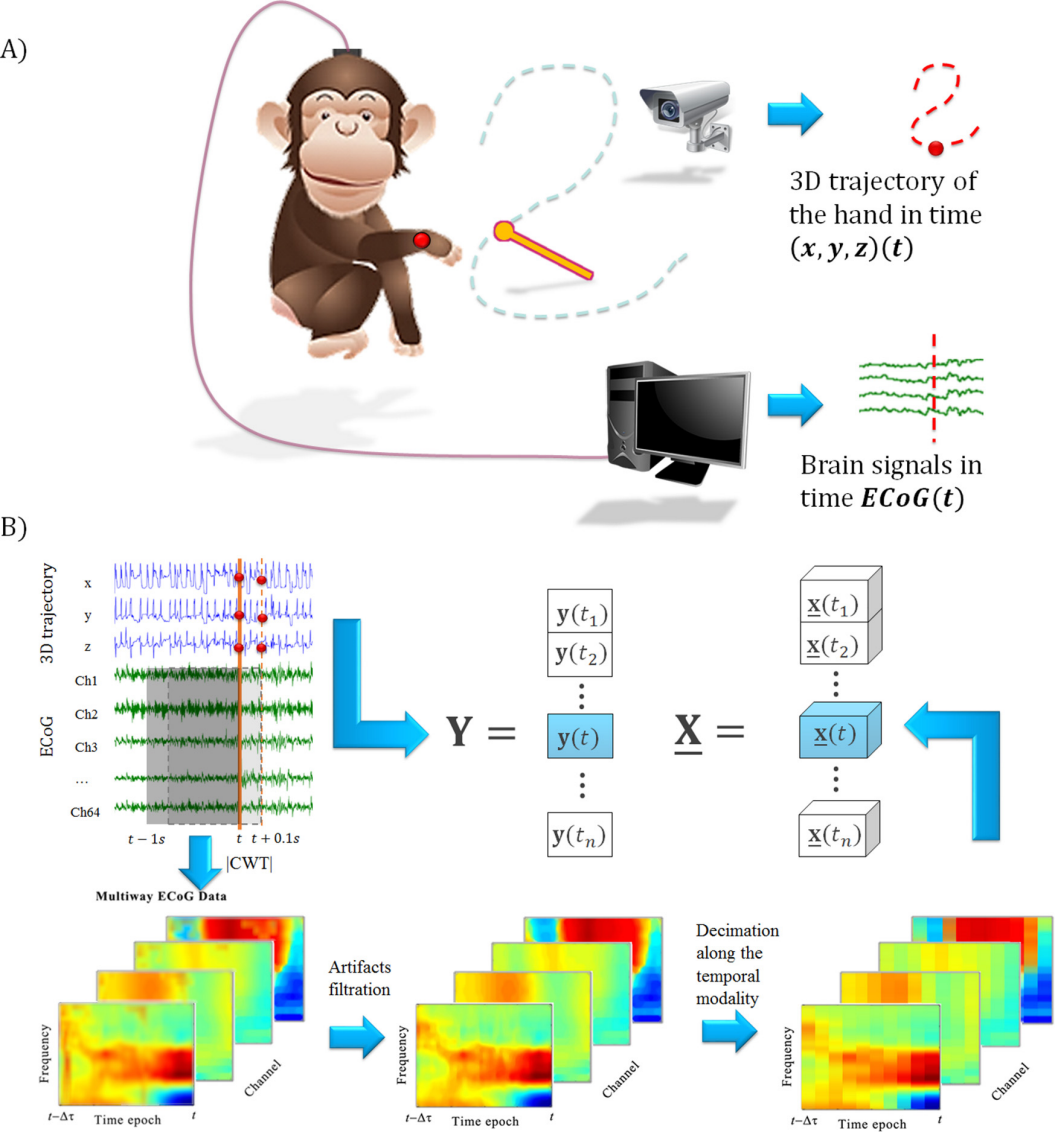
Experimental scheme and data formation. (A) The scheme of the experiment. The monkey is following the food with its hand. 3D coordinates of the hand are recorded simultaneously with monkey ECoG brain activity [22]. (B) For each moment *t*, the response variable y(*t*) is formed from the 3D coordinates of the monkey’s hand at this moment. To form the explanatory variable x(*t*), the ECoG epoch [*t*–Δ*τ*, *t*], Δ*τ* = 1 s is mapped by the Continuous Wavelet Transform (CWT). Then, the logarithm of the absolute values of the wavelet coefficients is taken, the procedure of the artifact filtration is applied, and the tensor is decimated along the temporal modality 100 times (1000 points to 10 points). Then the whole procedure is repeated for the next time moment *t*_*i*_ + 1 = *t*_*i*_ + Δ*t*, Δ*τ* = 0.1 s.

The publicly available dataset recorded and distributed by the Laboratory for Adaptive Intelligence, BSI, RIKEN (http://neurotycho.org) was used to evaluate the methods. During the experiments, the 3D position of the monkey’s right hand was recorded simultaneously with the epidural ECoG signal of the brain [26, 77]. The dataset used in the current paper consists of 20 recordings, corresponding to two Japanese macaques (10 recordings of each monkey), denoted as ‘B’ and ‘C’ in the database [22]. The monkeys were trained to receive pieces of food with their right hands. The pieces were demonstrated by the experimenter at random locations at a distance of about 20 cm from the monkeys. The new trial started when the monkey finished eating the previous piece of food. The ECoG data were acquired with 64 electrodes (Blackrock Microsystems, Salt Lake City, UT, USA) implanted in the epidural space of the left hemisphere (see Fig 1). A sampling rate of 1000 Hz per channel was provided. To record the hand trajectories, an optical motion capture system was used (Vicon Motion Systems, Oxford, UK) with a sampling rate of 120 Hz. The experiment scheme is represented in Fig 2A. Additional description of the experiments and analyzed data is given in [22, 77].

The duration of each of 20 experiment sessions was 15 minutes. The first 10 minutes of each recording were used for model identification and the last 5 minutes were employed for testing of the corresponding model.

#### Feature extraction

The input data tensor **X** was formed from the ECoG epochs. Each epoch contains 1 second of the signal, mapped to the temporal-spatial-frequency space by means of the wavelet transform. The complex Morlet wavelet was chosen as a mother wavelet due to its widespread application for BCI data analysis [22, 26, 30, 40, 46]. The epochs were taken continuously with time shifts equal to 100 ms. Based on [29], frequencies from 10 to 150 Hz with a step of 10 Hz were chosen. The logarithm of the absolute values of the wavelet coefficients was taken. After formation of the input tensor **X**, the chewing artifacts [22] were filtered in the way described in [29]. Then, the feature tensor was decimated along the temporal modality 100 times, by averaging the data in 10 sliding windows of 100 ms length. A detailed description of the analyzed data formation procedure is given in [29]. The matrix of the response variables **Y** was formed from the corresponding 3D coordinates of the monkey’s hand. Whereas for PLS, NPLS, SNPLS, PNPLS methods matrix **Y** represents 3D coordinates, the Kalman Filter uses the extended matrix of the response variables which additionally includes velocity and acceleration **Y**_*KF*_ = [**Y**, **Y**', **Y**'']. The explanatory variables tensor **X** is identical for all the methods. Fig 2B represents the general scheme of the data preparation procedure.

To validate the methods, the explanatory variables tensor **X** and the matrix of the response variables **Y** were split into training (70%) and test (30%) subsets for each recording:

$$\text{for training data}\;{\left\{{\underline{X}}_{\text{training}}\in {ℜ}^{6021\times 15\times 10\times 64},\;\begin{array}{c} Y_{\text{training}}\in {ℜ}^{6021\times 3},\;\text{for PLS-family methods}\\ \left[Y_{\text{training}},Y_{\text{training}}^{\prime },Y_{\text{training}}^{\prime \prime }\right]\in {ℜ}^{6021\times 9},\;\text{for KF} \end{array}\right\}}_{i=1}^{20},$$ (16)

$$\text{for test data}\;{\left\{{\underline{X}}_{\text{test}}\in {ℜ}^{2580\times 15\times 10\times 64},\;\begin{array}{c} Y_{\text{test}}\in {ℜ}^{2580\times 3},\;\text{for PLS-family methods}\\ \left[Y_{\text{test}},Y_{\text{test}}^{\prime },Y_{\text{test}}^{\prime \prime }\right]\in {ℜ}^{2580\times 9},\;\text{for KF} \end{array}\right\}}_{i=1}^{20}.$$ (17)

#### Parametrization

Three state-of-the-art methods (Kalman Filter, generic PLS, and tensor-based NPLS) and two proposed tensor-based approaches (SNPLS and PNPLS) were compared using a set of optimized parameters. The optimal number of factors *F* (PLS, NPLS, SNPLS, PNPLS) as well as the smoothing parameter *λ* (SNPLS, PNPLS) were chosen on the training set by the 10-fold cross-validation procedure [64] for each recording. The preliminary study on one recording gave the polynomic parameters for the PNPLS algorithm: the number of points chosen for the polynomial coefficients estimation was equal to *L* = 2*l* + 1 = 9 and the polynomic power was *p* = 2. Then, these parameters were fixed for all other recordings. Similarly, the optimal derivative order chosen for the SNPLS approach was *s* = 3.

#### Results

**Fig 3:**
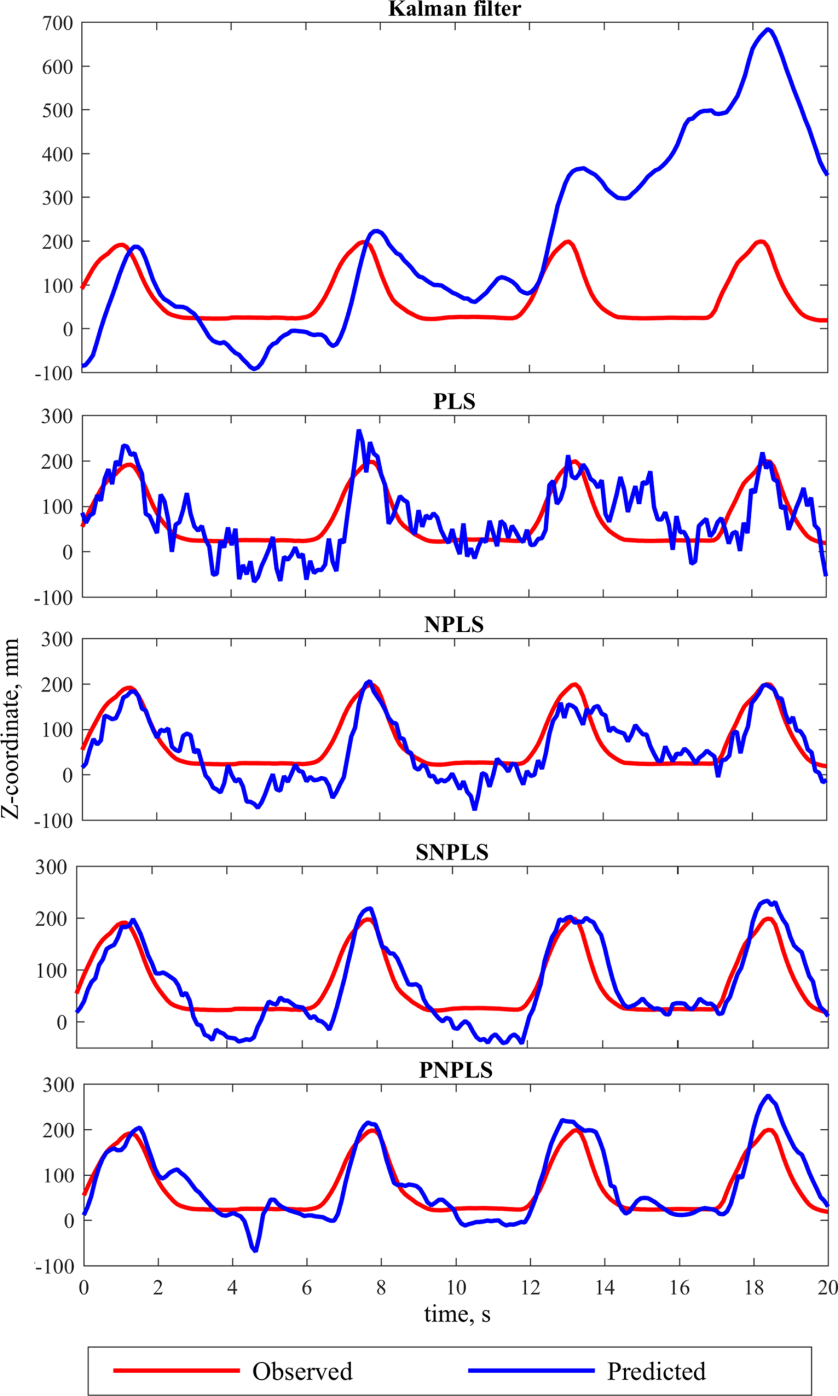
Example of the observed and predicted data. Examples of the observed and predicted (Kalman Filter, PLS, NPLS, PNPLS, and SNPLS) trajectories of Z-coordinate of the monkey’s wrist.

The models were calibrated separately on each training recording and tested on the corresponding testing data. An example of the application of the compared methods to the same interval of data is illustrated in Fig 3.

**Fig 4:**
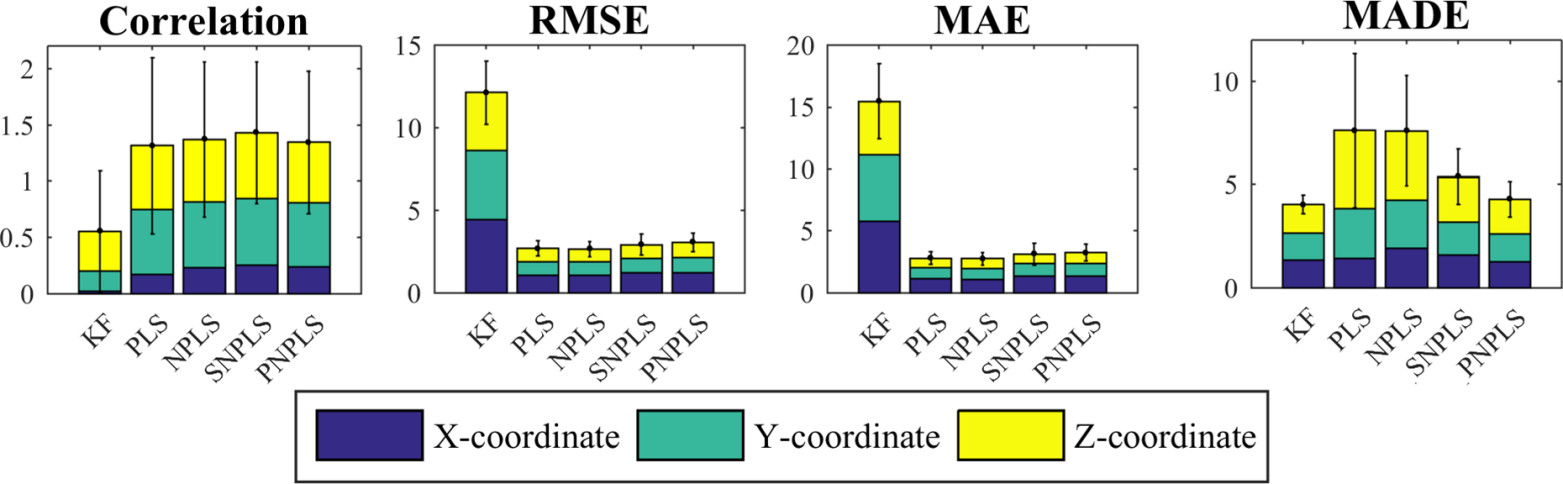
Prediction quality characteristics. The criteria of prediction quality, estimated for 3D trajectories for the models given by KF, PLS, NPLS, SNPLS, and PNPLS. The results are averaged over 20 recordings for two monkeys.

Fig 4 presents the criteria for comparing the methods’ performance (Pearson correlation, RMSE, MAE, MADE) for the Kalman Filter, PLS, NPLS, SNPLS, and PNPLS, averaged over all the recordings. Table 1 represents the *p*-values for all the pairs of the methods. In the present study, the significance level of *α* = 0.05 is chosen. The algorithms of the PLS family, and in particular the proposed SNPLS and PNPLS, significantly outperform the KF in correlation, RMSE, and MAE. The difference between the generic methods (PLS, NPLS) and the proposed approaches (SNPLS, PNPLS) for these criteria is insignificant (Table 1) and does not exceed 5% (Fig 4). In general, the presented prediction accuracy corresponds to the accuracy reported for this database [22, 26, 29, 46].

**Table 1:** *p*-values of the difference between the quality evaluation criteria of the methods (ANOVA test, significance level *α* = 0.05).

		*p*-value			
Compared methods		Correlation	RMSE	MAE	MADE
KF	SNPLS	0.000	0.000	0.000	0.000
	PNPLS	0.000	0.000	0.0l00	0.132
PLS	SNPLS	0.456	0.126	0.108	0.002
	PNPLS	0.848	0.007	0.010	0.000
NPLS	SNPLS	0.678	0.093	0.073	0.000
	PNPLS	0.853	0.005	0.005	0.000
SNPLS	PNPLS	0.529	0.409	0.548	0.000

KF, SNPLS and PNPLS demonstrated better smoothness than PLS and NPLS, which is reflected by the MADE criterion: the improvement is significant (Table 1) and varies from 30% to 47% (Fig 4). At the same time, although KF showed the best smoothness, the difference between KF and the proposed PNPLS approach is less than 6% and is insignificant.

**Fig 5:**
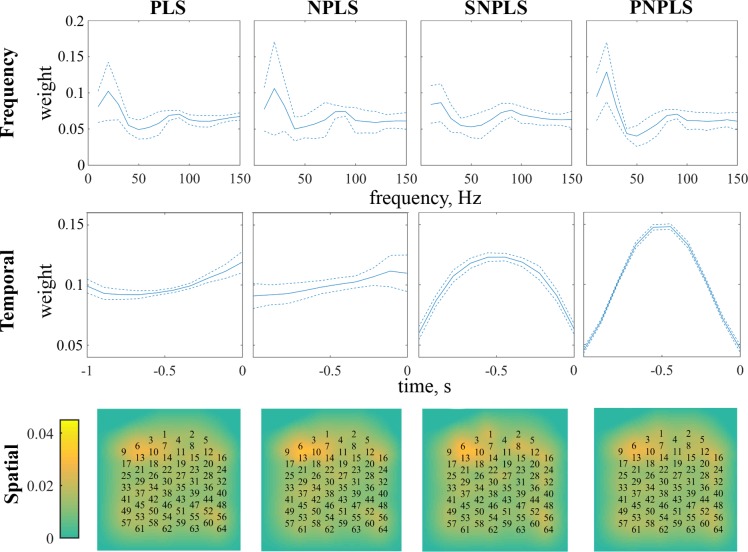
Modalities influences. The average influences of the elements of frequency, temporal, and spatial modalities on the prediction model for PLS-family methods (generic PLS, NPLS, SNPLS, and PNPLS) for monkey “B”.

To illustrate PLS-family predictive models, the averaged influence [78] of the frequency, temporal, as well as spatial modalities estimated for the monkey “B” are shown in Fig 5 (the results for the monkey “C” are consistent). For each modality, the influences of its elements are evaluated as the weights of sum of the absolute values of the related predictive model’s coefficients.

### Prediction delay comparison

System latency is an important characteristic for BCIs [55]. Smoothing generally introduce the delay to the prediction. An additional study was carried out to assess and compare prediction delays of the analyzed methods.

**Fig 6:**
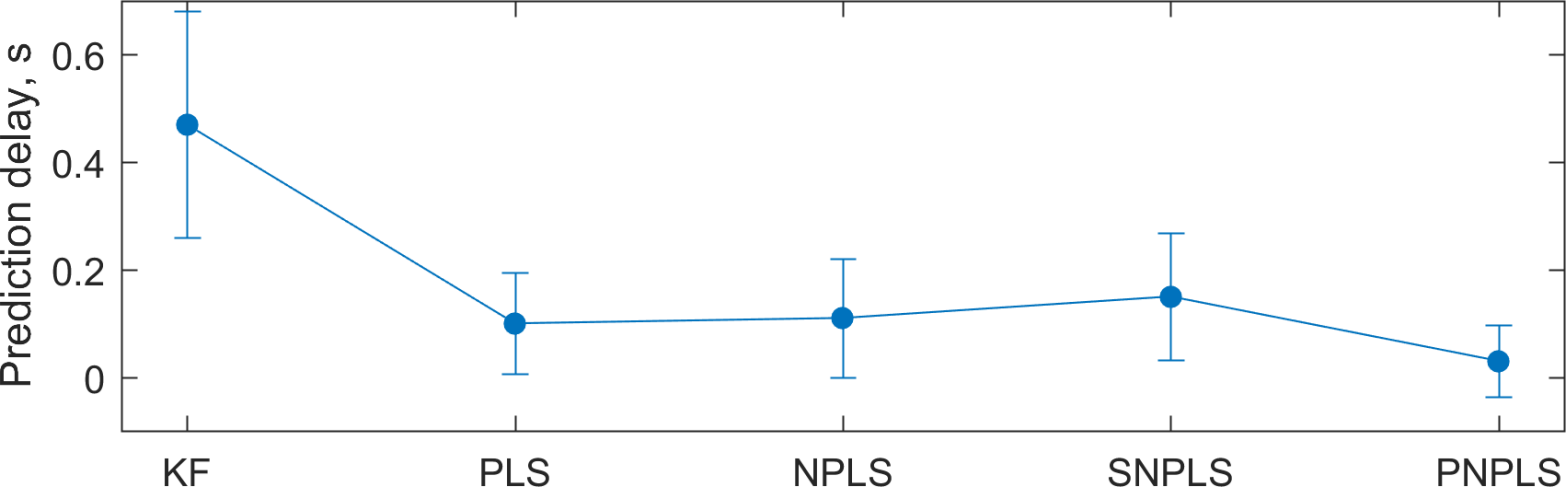
Prediction delays. Averages prediction delays of the Kalman Filter, PLS, NPLS, SNPLS, and PNPLS approaches. PLS, NPLS, SNPLS, and PNPLS significantly outperform KF, whereas the differences between PLS, NPLS, SNPLS, and PNPLS are not significant (significance level *α* = 0.05)

To compare latencies, the predicted trajectories generated by the analyzed approaches were shifted in time to maximize the correlation between predicted and observed trajectories. Prediction delays were estimated as minimal shifts of the predicted signals, which maximizes the correlation between predicted and tested trajectories. The shift interval *S* = [0, 2] s was studied. Fig 6 shows averaged delays for the analyzed methods over monkeys, sessions, and coordinates. The delay of the Kalman filter is 0.47±0.21 s. Significantly lower (*α* = 0.05) prediction delays are demonstrated by PLS-family algorithms (0.10±0.09 s, 0.11±0.11 s, 0.15±0.12 s, and 0.03±0.07 s for PLS, NPLS, SNPLS, and PNPLS, respectively). Differences between the PLS, NPLS, SNPLS, and PNPLS latencies are not significant.

## Discussion

Motor-related BCI systems are of great importance since they can help people with severe motor disabilities to recover their functionality. However, decoding the neural activity is a challenging task [1]. BCI control of external devices like wheelchairs, artificial arms, exoskeletons, etc. in real life is especially difficult, due to safety issues [24]. This imposes additional restrictions on BCI decoders, as well as on the smoothness of the predicted trajectories, in particular. The standard approach for improving smoothness consists of post-filtering of the obtained prediction [55, 79]. Such filters (e.g. sliding window averaging) bring a delay to the system response. More complex nonlinear filters, such as KF, are labor-consuming, which decreases the decision rate of real-time BCI systems, especially in the case of high dimensional data, and also introduces a prediction delay. Both a high decision rate and minimal system latency are essential requirements for the comfortable use of real-time BCI systems [55].

The methods proposed in the current paper, namely SNPLS and PNPLS, are based on multi-way data analysis, which is known to be efficient for neural data treatment [40, 47, 80]. These methods construct the decoders with the possibility of controlling the smoothness of the predicted trajectories. The proposed algorithms try to improve the smoothness of prediction without increasing the system latency. Penalization integrated into the linear decoder identification procedure can be interpreted, as the penalization of informative but noisy variables. The proposed methods improve solutions stability by penalizing unsmooth predictions without increasing the complexity of the optimization problem (sum-of-squares optimization). The proposed methods do not change the predictive model complexity as well. Solutions remains linear, and the resulted model can be efficiently applied for high dimensional data flow decoding in real-time on standard computer (Intel Dual Core, 3.16 GHz; RAM 3.25 Gb) [80]. The proposed methods could be applied in a wide range of applications beyond neuroscience.

To validate the efficiency of SNPLS and PNPLS for BCI tasks, the algorithms were compared with a set of state-of-the-art approaches. The Kalman Filter has been chosen as a method which generates a smooth prediction and is widely applied in BCIs. However, it contains the inversion of the matrix. This limits the application of the KF for high dimensional tasks [53]. Unlike the KF, the algorithms of the Partial Least Squares family are particularly suited for the high dimensional data and are also often used in BCI systems [22, 46]. Improvement of the prediction smoothness of these methods, with minimal loss of accuracy as well as supplementary delay, was the particular goal of the article. The prediction accuracy of the compared methods was evaluated by means of Pearson correlation, RMSE, and the more robust to outliers MAE criteria. To evaluate the smoothness, the MADE criterion was chosen. It combines the robustness of the *L*_1_-norm and allows assessment of the smoothness.

The algorithms of the PLS family significantly outperform the KF in prediction accuracy estimated with Correlation, RMSE, MAE, whereas the difference among the PLS-based approaches is insignificant. Due to matrix inversion, KF could be more sensitive to high dimensional and noisy explanatory variable in comparison to PLS-family algorithms. Additional pre- and/or post-processing of data or Kalman algorithm’s modifications could improve its prediction accuracy for the current task and this is the subject for future researches.

From the smoothness point of view, KF, PNPLS, and SNPLS significantly outperform generic PLS and NPLS. KF demonstrates the best smoothness. However, the results of KF and new methods are comparable. Thus, PNPLS and SNPLS algorithms combined the prediction accuracy of the PLS-family algorithms with the smoothness approaching the KF. In addition, SNPLS and PNPLS provide lower prediction delay in comparison with the KF. The KF approach latency reaches half a second (470±210 ms) that could be noticeable and irritable for subjects. As it was reported in [79] for spikes decoding methods in monkeys experiments, whereas delays up to 200 ms provide minimal influence, every 100 ms of additional delay added about 200 ms to the reaching time. The studies in human also demonstrated that delays about 200 ms slow down reaching tasks and decrease accuracy [81]. For simulated human experiments, increasing of average reaching time by over 5 s due to visual feedback delay of 300 ms is reported in [55]. Contrary to the KF, the PLS, NPLS, SNPLS, and PNPLS methods provide a latency of about 100 ms that is equal to the decision rate of the system. However, additional real-time closed-loop BCI experiments should be carried out in human to clarify the latency’s tolerable level.

Generally, criteria values appropriated for the practical applications are derived according to the subject satisfaction in practical experiments. The influence of different parameters such as error magnitude, processing delays, etc. on closed-loop BCI in human was studied, in particular in [55]. The comparison of decoding approaches during the clinical closed-loop BCI trials will be the next step of our research.

Fig 5 demonstrates the modality influence analysis results [78] for the PLS-family methods, in frequency, spatial and temporal domains. In the frequency modality, frequencies influence distributions are similar for all the considered approaches. The distributions have two maximums around 20 Hz and 90 Hz. In the temporal modality, the PLS and NPLS models tend to give close weights to all elements of the analyzing interval, with the maximums near 0 moments. Contrarily, coefficient distribution of the SNPLS and PNPLS models have maximums in the middle of the analyzing intervals. It is possible that weight shapes are responsible for the predicted trajectories smoothness. On the other hand, prediction delay evaluation demonstrated that there is no additional latency due to these weights distributions, as one might expect. Additional study is needed to clarify this effect. In the spatial modality, electrode weights are comparable for all the considered approaches. The most informative electrodes (6, 10, and 13) are the same for all the methods. This could be explained by detecting the readiness potentials, i.e. cortical contribution to the pre-motor planning of volitional movement in the supplementary motor area. Additional information about the readiness potentials could be found in [82].

The influences analysis allows estimating the stability of the prediction models by means of assessment of the weights variability in each modality. Standard deviations through all the modalities are *σ*_PLS_ = 0.005±0.005, *σ*_NPLS_ = 0.005±0.008, *σ*_SNPLS_ = 0.005±0.005, *σ*_PNPLS_ = 0.004±0.005. The differences of the standard deviations are not significant (*α* = 0.05). Thus, the proposed PNPLS and SNPLS methods keep the robustness of the generic PLS and NPLS approaches.

Comparing the proposed methods, both SNPLS and PNPLS allow improving of the smoothness of the predicted trajectories. The PNPLS prediction smoothness surpasses the SNPLS one, while the prediction latencies for both approaches are comparable. On the other hand, SNPLS provided slightly a better accuracy (Fig 4). The advantage of SNPLS is that it depends on fewer external parameters than PNPLS, thus it is preferable in the case of restricted computation time for model identification.

### Limitations of the present study and future work

The main limitation and disadvantage of the proposed approaches is the time-consuming estimation of a set of the external parameters. The smoothness parameter, the polynomial parameters for PNPLS, the derivative order for SNPLS, as well as the number of factors for SNPLS and PNPLS, need to be evaluated. In this study, a time-consuming cross-validation procedure is applied to identify the external parameters of the algorithms. Additional investigation is needed to optimize parameters estimation procedure.

Parametrization and calibration is the offline procedure. Generally, recalibration is not required for ECoG-based BCIs during a long period [80]. Nevertheless, the fast online calibration would be a strong point for the BCI system. Recently, the Recursive NPLS algorithm was proposed [46] for adaptive BCI system calibration. The combination of the SNPLS and PNPLS methods with RNPLS will allow online adjustment of the decoder to the non-stationary brain activity.

As it was reported in [49, 83], methods that demonstrate good performance for offline decoding could be less efficient for online applications, while other methods provide better performance in closed-loop conditions. The next step of the study will be closed-loop application and the evaluation of decoding approaches, and in particular the application of the algorithms in clinical BCI to calibrate the BCI decoder. The fully-implantable device WIMAGINE^®^ [25] for chronic measurement and wireless transmission of ECoG data, as well as the full body exoskeleton EMY^®^ [84], are currently developed within the framework of the CEA-LETI-CLINATEC^®^ BCI project. Clinical trials of the exoskeleton control by tetraplegic subjects is in preparation.
